# Highly water pressurized brown rice improves cognitive dysfunction in senescence-accelerated mouse prone 8 and reduces amyloid beta in the brain

**DOI:** 10.1186/s12906-018-2167-z

**Published:** 2018-03-27

**Authors:** Michiaki Okuda, Yuki Fujita, Takuya Katsube, Hiromasa Tabata, Katsumi Yoshino, Michio Hashimoto, Hachiro Sugimoto

**Affiliations:** 1 Graduate School of Brain Science, Doshisha University, 4-1-1 Kizugawadai, Kizugawa, Kyoto, 619-0225 Japan; 2 Shimane Institute for Industrial Technology, 1 Hokuryo-cho, Matsue, Shimane 690-0816 Japan; 3 Faculty of Medicine, Shimane University, 89-1 Enya-cho, Izumo, Shimane 693-8501 Japan; 4 Present address: Green Tech Co. Ltd, 2–1-8 Horidome-cho, Chuo-ku, Tokyo, 103–0012 Japan

**Keywords:** Alzheimer’s disease, Amyloid-β, Brown rice, Food engineering, High-pressure treatment

## Abstract

**Background:**

Alzheimer’s disease (AD) is the most common form of dementia and the number of AD patients continues to increase worldwide. Components of the germ layer and bran of Brown rice (BR) help maintain good health and prevent AD. Because the germ layer and bran absorb little water and are very hard and difficult to cook, they are often removed during processing. To solve these problems, in this study, we tried to use a high-pressure (HP) technique.

**Methods:**

We produced the highly water pressurized brown rice (HPBR) by pressurizing BR at 600 MPa, and then we fed it to an AD mouse model, senescence-accelerated mouse prone 8, to investigate the therapeutic effects of HPBR on cognitive dysfunction by Y-maze spatial memory test.

**Results:**

HP treatment increased the water absorbency of BR without nutrient loss. HPBR ameliorated cognitive dysfunction and reduced the levels of amyloid-β, which is a major protein responsible for AD, in the brain.

**Conclusions:**

These results suggest that HPBR is effective for preventing AD.

## Background

Alzheimer’s disease (AD) is a neurodegenerative disease, and of the estimated 44 million people with dementia worldwide, more than half have AD [1]. The currently available drugs for AD such as the acetylcholinesterase inhibitors, donepezil, rivastigmine, and galantamine, and the N-methyl-d-aspartate receptor antagonist memantine are limited only to symptomatic treatment [2]. Prevention measures are thus highly desirable. In the present study, we have analyzed the benefits of brown rice (BR, *Oryza sativa* L.) for the prevention of AD and other dementias because it is widely cultivated and consumed in Asia and other regions. BR is generally processed to remove the germ layer and bran because they are very hard, difficult to cook, and poorly digested. However, these regions contain many ingredients that can be used to prevent AD and other dementias. For example, ferulic acid (FA) has antioxidant [3] and neuroprotective effects [4], and inhibits amyloid β (Aβ) production [5] and aggregation [6]. They also include vitamin B6, vitamin B12 and folic acid, which are essential for methionine metabolism; the deficiency of them leads to an increase in blood homocysteine levels, increasing the risk of vascular diseases [7] and AD [8]. The levels of gamma-aminobutyric acid (GABA), which acts as an excitatory neurotransmitter in the brain and plays a crucial role in regulating nerve excitation, are reduced in the brains of AD patients than in the brains of healthy adults [9].

In this study, highly water pressurized brown rice (HPBR) was produced to increase the palatability and nutrient availability of BR. High-pressure (HP) treatment is a non-thermal food processing technique and has been used in the field of food processing and other fields [10]. HP has a powerful bactericidal effect and improves food storage life [11]. HP treatment physically disrupts cells and connective tissue [12] and chemically denatures proteins [13], starch [14] and lipids [15]. Unlike heat treatment, HP treatment does not disrupt covalent bonds, thus it improves the digestion and absorption of foods without nutrient loss. It also lowers the allergen content [16] and maintains food quality by enzyme inactivation [17]. The nutritional value of HPBR was thus maintained following treatment, and its water absorbance was increased. The effects of HPBR were evaluated in an AD model, senescence-accelerated mouse prone 8 (SAMP8). SAMP8 is a substrain of the senescence-accelerated mouse (SAM) line, which is a murine model of accelerated aging [18], and has been widely used to evaluate the efficacy of drug candidates for AD and beneficial foods [19–24]. SAMP8 mice exhibit age-related memory and learning deficits [25, 26] and develop AD-like Aβ pathology in the brain [27]. Their symptoms are similar to those of sporadic, age-related AD rather than familial, inherited AD [28]. Because most AD cases are sporadic and not familial, the SAMP8 mice was considered more suitable than other transgenic mouse models overexpressing genes with familial AD mutations. HPBR ameliorated cognitive dysfunction and reduced Aβ levels in the brain, but polished rice (PR) and wheat did not, suggesting that HPBR is effective for preventing AD and other dementias.

## Methods

### Materials

HPBR, BR, and PR were obtained from Elise Co., Ltd. (Iinan-cho, Japan) and were all cultivated at Iinan-cho, Shimane, Japan, and produced from the same variety of rice. Wheat powder for the control group was purchased from Nisshin Seifun Inc. (Tokyo, Japan). HPBR was prepared from BR by exposing it to water at a hydrostatic pressure of 600 MPa for 5 s using a hydrostatic pressurizer (Elise Co., Ltd.).

### Analysis of nutritional composition

The basic nutrient composition of HPBR, BR, and PR was estimated at Shimane Environment & Health Public Corporation (Matsue, Japan) and Shimane Institute for Industrial Technology (Matsue, Japan). The energy content was estimated by the Atwater method. The amount of water was estimated by drying in a vacuum method. The amount of protein was estimated by the Kjeldahl method. The amount of lipids was estimated by the acid digestion method. The amount of carbohydrate was estimated by the subtraction method. The amounts of soluble fiber and insoluble fiber were measured by the Prosky method. The amounts of sodium and calcium were estimated by the atomic absorption. The amounts of vitamin B and niacin were measured by a microbiological assay. The amount of FA and GABA were assayed by high-pressure liquid chromatography. The analyses were conducted using the analytical methods recommended by the Consumer Affairs Agency, Government of Japan [29] and the Analytical Manual of Standard Tables of Food Composition in Japan [30]. Standard for the amount of FA in PR and BR were obtained from Nishizawa et al. [31] and the other data was obtained from the Standard Tables of Food Composition in Japan [32].

### Water absorbency test

The water absorbed by HPBR was considered as a measure of its cooking characteristics. Two grams each of HPBR, PR, and BR was soaked in water for 1 h, after which each sample was weighed. Then, after removing the excess water, the mean amount of water absorbed by HPBR, PR, and BR was recorded as the water absorbency.

### Animals and treatments

Twenty- six 3-month-old male SAMP8/TaSlc mice were obtained from Japan SLC Inc. (Hamamatsu, Japan) and maintained in a regulated environment at 24 ± 2 °C, 50 ± 10% humidity, and a 12-h inverted light–dark cycle. The mice were allowed free access to food and tap water. Each of HPBR, PR and wheat were powdered and mixed 50% with MF diet (Oriental Yeast Co., Ltd., Tokyo, Japan) and orally administered to the mice for 2 months (9 weeks). The mice were equally divided into three groups of eight or nine animals based on body weight and performance in the Y-maze test. All experimental procedures involving mice and their care were conducted in accordance with the ethical guidelines of the Kyoto University Animal Experimentation Committee and the guidelines of the Japanese Pharmacological Society (approved number: 2015–13).

### Y-maze test

Working memory was evaluated using the Y-maze test at the start of the administration and then once each month. The mice were placed at one of the three arms of the Y-maze, which were 30 cm in length with equal angles between them. The mice were allowed to move freely in the maze for 8 min, and the sequence and number of arm entries were recorded. Spontaneous alternation behavior was used as a measure of spatial memory and defined as entry into all three arms as consecutive choices. The percentage of spontaneous alternations was calculated as follows:

$$ Number\ of\ spontaneous\ alternation/\left( number\ of\ total\  arm\  entries-1\right)\kern0.87em \times \kern0.5em 100 $$

### Motor function tests

Coordinated movements of mice were evaluated on a rotarod apparatus (MK-670, Muromachi Kikai Co., Ltd., Tokyo, Japan) at the end of the administration. At first, the mice were placed on a rotating rod that was accelerated to 8 rounds per minute for 60 s in order to accustom them to the rotating rod. And then the mice were placed again on the rod that was accelerated to 40 rounds per minute in 300 s, and the time until falling from the rod was recorded for a maximum of 300 s. The mice were tested twice, and the mean time was recorded as their score. Grip strength of the mice was measured using a grip strength meter (MK-380, Muromachi Kikai Co., Ltd.). Individual mice were placed on the metal mesh of the meter, and the tail was pulled back horizontally. The grip strength of each mouse when it could no longer hold on to the mesh was recorded. Grip strength was recorded as the mean of three trials.

### Protein extraction from the brain tissue and measure amyloid-β

After the administration period, the mice were sacrificed by the induction of deep anesthesia with an intraperitoneal induction of 100 mg/kg sodium pentobarbital (Kyoritsu Seiyaku Corp., Tokyo, Japan). The whole brain was excised and homogenized in 10 volumes (*w*/*v*) of 2 × radioimmunoprecipitation assay buffer (Nacalai Tesque, Inc.) with 0.5 mM phenylmethylsulfonyl fluoride (Sigma-Aldrich Corp. St. Louis, MO, USA), 1 × PhosSTOP (Roche, Basel, Switzerland), and 1% protease inhibitor cocktail (Nacalai Tesque, Inc.).

To measure Aβ, the homogenate was centrifuged at 15000×g at 4 °C for 20 min and the supernatant was collected as the protein extract. The amount of Aβ_1–42_ and Aβ_1–40_ in the brains was determined using an enzyme-linked immunosorbent assay kit for Aβ (Wako Pure Chemical Industries, Inc.) and a microplate reader (Model 680; Bio-Rad Laboratories, Inc.), according to the manufacturer’s instructions. The protein extract was diluted 10-fold with the dilution buffer provided in the kit. The level of amyloid precursor protein (APP) in the brain was estimated western blotting. The protein extract was mixed with an equal amount of Tris-SDS β-mercaptoethanol sample buffer (Cosmo Bio Co., Ltd., Tokyo, Japan) and boiled at 100 °C for 10 min. The 10 μL aliquots of each sample were electrophoresed at 200 V for 1 h on a 5%–20% polyacrylamide gel (Wako Pure Chemical Industries Ltd.). After separation, the proteins were transferred onto a 0.45 μm polyvinylidene difluoride membrane (Merck Millipore), which was run at 15 V for 30 min. After blocking with 2.5% skim milk (Nacalai Tesque, Inc.) in TBS-T (Sigma-Aldrich Corp.) for 1 h, the blots were incubated with anti-APP (1:200 dilution; Immuno-Biological Laboratories Co., Ltd., Fujioka, Japan) or anti-actin (1:5000 dilution, Cell Signaling Technology, Inc. Danvers, MA, USA) primary antibodies at 4 °C overnight. The blots were washed thrice with TBS-T for 10 min before incubation with HRP-conjugated anti-rabbit immunoglobulin G secondary antibody (1:3000 dilution; GE Healthcare Little Chalfont, Buckinghamshire, UK) for 2 h at room temperature and washed thrice with TBS-T for 10 min. APP and action were detected using Chemi-Lumi One (Nacalai Tesque, Inc.), and the protein bands were analyzed with an ImageQuant LAS4000 (GE Healthcare) reader. The band density of APP was corrected by comparing it with the band intensity of actin.

### Data analysis

Y-maze test results and body weights were analyzed by two-way analysis of variance (ANOVA) and the Bonferroni post-test. Other data were analyzed by one-way ANOVA and Dunnett’s post-tests. Graphpad Prism (GraphPad Software Inc., San Diego, CA, USA) was used for statistical analysis, and *P* <  0.05 was considered statistically significant.

## Results

### Nutrient composition

The nutrient composition of PR, BR and HPBR is shown in Table 1. The amount of water, carbohydrate, and protein did not differ. However, there was more dietary fiber and larger amounts of minerals, vitamins, FA, and GABA in BR than in PR, suggesting that BR is more complete and balanced than PR. The nutrition composition of BR and HPBR did not differ, suggesting that HP treatment does not break down any nutrients.

**Table 1:** Nutrient composition (per 100 g) in polished rice (PR), brown rice (BR) and highly water pressurized brown rice (HPBR)

	PR	BR	HPBR
Energy (kcal)	358	353	356
Water (g)	14.9	14.9	13.1
Carbohydrate (g)	77.6	74.3	76.8
Protein (g)	6.1	6.8	7.7
Lipids (g)	0.9	2.7	2.0
Soluble fiber (g)	< 0.1	0.7	4.1
Insoluble fiber (g)	0.5	2.3	3.0
Sodium (mg)	1.0	1.0	3.0
Calcium (mg)	5.0	9.0	9.0
Vitamin B1 (mg)	0.08	0.41	0.41
Vitamin B6 (mg)	0.12	0.45	0.32
Niacin (mg)	1.2	6.3	7.5
Ferulic acid (mg)	9.4	41.8	27.0
GABA (mg)	1.5	7.0	9.1

### HP treatment improves water absorbency

**Fig. 1:**
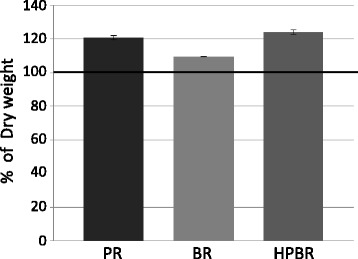
Comparison of water absorbency. PR: polished rice, BR: brown rice, HPBR: highly water pressurized brown rice. Mean ± SD, *n* = 3

The water absorbencies of PR, BR, and HPBR, a measure of cooking property, are shown in Fig. 1. After soaking in water for 1 h, the weights of PR, BR, and HPBR increased by 20.5% ± 1.3%, 9.3% ± 0.3%, and 24.0% ± 1.3%, respectively. The higher water absorbency of HPBR suggests that it would be easier to cook HPBR than BR and PR.

### HPBR administration ameliorates cognitive dysfunction in SAMP8 mice

**Fig. 2:**
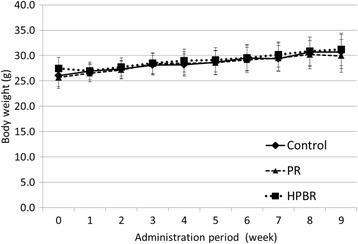
Change in body weight during the administration. PR: polished rice, HPBR: highly water pressurized brown rice. Mean ± SD, *n* = 9 (control and HPBR groups) or 8 (PR group)

HPBR was orally administered to 3-month-old male SAMP8 mice for 2 months with wheat (control) or PR as the comparison groups. There were no differences in the average weights between the groups (Fig. 2).

**Fig. 3:**
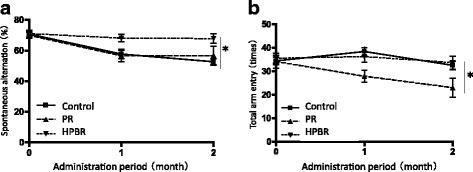
Y-maze test. **a** Spontaneous alternation behavior rate. *P* = 0.009, two-way ANOVA, *P < 0.05, control vs. HPBR, Bonferroni post-test. **b** Total arm entry. *P* = 0.04, two-way ANOVA, *P < 0.05, PR vs. HPBR or control, Bonferroni post-test. PR: Polished rice, HPBR: highly water pressurized brown rice. Mean ± SE, *n* = 9 (control and HPBR groups) or 8 (PR group)

Spatial working memory of the mice was evaluated by Y-maze tests (Fig. 3), which showed that at the start of feeding, spontaneous alternations occurred with a frequency of approximately 70% in all groups (70.8% ± 1.9% in the control group, 70.8% ± 2.0% in the PR group, and 70.8% ± 2.2% in the HPBR group; Fig. 3a). At 9 weeks, percentage of spontaneous alternations in the control and PR groups decreased to 52.6% ± 2.4% and 49.5% ± 8.8%, respectively, but remained unchanged at approximately 70% (67.8% ± 3.0%) in mice fed with HPBR (*P* = 0.009, two-way ANOVA; *P* <  0.05 control group vs. HPBR group, Bonferroni post-test). These results indicate that HPBR ameliorates cognitive dysfunction in SAMP8 mice. The total number of arm entries decreased with age in the PR group, not in the control and HPBR groups. The number of total arm entries at 9 week of feeding was 32.6 ± 1.8 in the control group, 26.3 ± 2.9 in the PR group, and 33.7 ± 2.9 in the HPBR group (Fig. 3b). The differences between the PR group and the other groups were significant (*P* = 0.04, two-way ANOVA; *P* < 0.05, PR group vs. control or HPBR group, Bonferroni post-test).

### HPBR also ameliorates motor dysfunction in SAMP8 mice

**Fig. 4:**
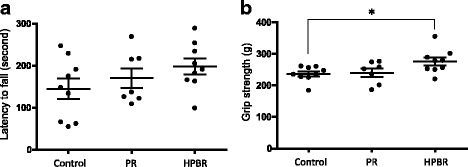
Evaluation of motor function. **a** Rota rod test. *P* = 0.24, one-way ANOVA. **b** Grip strength test. *P* = 0.04, one-way ANOVA and *P < 0.05, control vs HPBR, Dunnett’s post-test. PR: Polished rice. HPBR: highly water pressurized brown rice. Mean ± SE, *n* = 9 (control and HPBR groups) or 8 (PR group)

A decline in motor function, measured by hand grip strength and walking ability, is associated with an increased risk of AD [33]. The walking ability and grip strength of the mice after the feeding period are shown in Fig. 4. In the rotarod test, the interval until falling was 37% longer in the HPBR group than in the control group, but the difference was not significant. (*P* = 0.24, one-way ANOVA, Fig. 4a). In grip strength test, the HPBR group had a significantly stronger grip strength than the control group (*P* = 0.04, one-way ANOVA; *P* < 0.05 control group vs. HPBR group, Dunnett’s post-test, Fig. 4b). The results suggest that HPBR ameliorates aging-related motor dysfunction.

### HPBR reduces the amount of Aβ_1–42_ in the SAMP8 mice brain

**Fig. 5:**
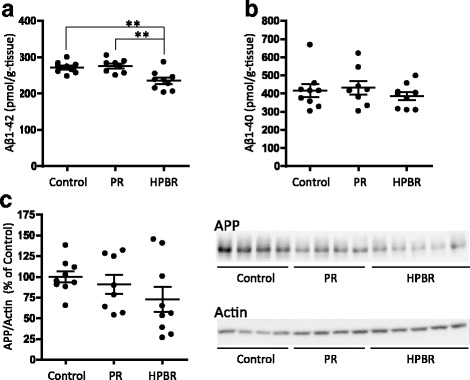
The amounts of Aβ and APP in the brain. **a** Aβ_1–42_. *P* = 0.004, one-way ANOVA and **P < 0.01, HPBR vs. control or PR, Dunnett’s post-test. **b** Aβ_1–40_. *P* = 0.54, one-way ANOVA. **c** APP and its representative image of western blotting. *P* = 0.26, one-way ANOVA. PR: Polished rice, HPBR: highly water pressurized brown rice. Mean ± SE, *n* = 9 (control and HPBR groups) or 8 (PR group)

To clarify the mechanism of improved cognition in SAMP8 mice by HPBR, we measured the amount of Aβ in the mice brain after HPBR administration (Fig. 5). The amount of Aβ_1–42_ in the HPBR group (235 ± 9 pmol/g of tissue) was significantly less than those in the control (272 ± 5 pmol/g of tissue) and PR groups (276 ± 9 pmol/g of tissue) (*P* = 0.004, one-way ANOVA; *P* < 0.01 HPBR group vs. control or PR group, Dunnett’s post-test, Fig. 5a). In contrast, there were no significant differences in the amount of Aβ_1–40_ among the groups (*P* = 0.54, one-way ANOVA, 416 ± 36 pmol/g of tissue in the control group, 435 ± 35 pmol/g of tissue in the PR group, and 386 ± 22 pmol/g of tissue in the HPBR group, Fig. 5b). The results indicate that the reduction of Aβ_1–42_ in the HPBR group result in the observed improvement of cognitive dysfunction (Fig. 3). The brain level of APP in the HPBR group was 27% less than that of the control group (Fig. 5c), but the difference was not significant (90.9% ± 11.7% of the control group in the PR group, and 73.0% ± 15.1% of the control group in HPBR group, *P* = 0.26, one-way ANOVA,).

## Discussion

The incidence of dementia, including AD, continues to increase worldwide [1]. Aβ is considered to be the major cause of AD and the large aggregates of Aβ, which is called senile plaque, is a major hallmark of AD. However, most Aβ cascade blockers such as β/γ-secretase inhibitors and Aβ immunotherapies have failed to show efficacy in clinical trials. As the development of Aβ abnormalities precedes the onset of cognitive dysfunction in AD by approximately 20 years [34], it is probably too late to provide these drugs and therapies after the onset of cognitive dysfunction. Therefore, preventive approaches including items in the daily diet are very important practical measures and we focused on BR. Although BR is nutrient-rich, containing many components effective against the development of dementia, it is very difficult for them to be absorbed them into the body because BR is difficult to digest. Thus BR was treated with HP to improve digestibility and absorption without changing its nutrient composition. HP can enhance the nutritional values of BR. For example, GABA in BR increases by HP treatment [35], and this agrees with our result. BR is difficult to cook because the bran is very hard and has decreased water absorbency. However, HPBR had the same level of high water absorbency as PR (Fig. 1). These results suggest that HPBR contained the high nutritional content of BR along with the good cooking characteristics of PR.

In the present study, we tested the effects of HPBR in vivo using the SAMP8 model. The HPBR-fed mice showed a higher rate of spontaneous alternation in the Y-maze test than the wheat-fed controls and PR-fed mice (Fig. 3a). The amount of Aβ_1–42_ in the brain of HPBR-fed mice was also lesser than those in the other groups (Fig. 5a). The results suggest that HPBR ameliorated the cognitive dysfunction of SAMP8 mice via the reduction of the amount of Aβ_1–42_. On the other hand, the amounts of Aβ_1–40_ among the groups did not differ (Fig. 5b). Aβ_1–42_ is more neurotoxic and easy to aggregate compared to Aβ_1–40_ [36, 37]. HPBR might specifically inhibit the production of Aβ_1–42_, as some nonsteroidal anti-inflammatory drugs and γ-secretase modulators do [38], but further research is needed to clarify the detailed mechanism. An increase in the amount of APP has been demonstrated in the brain of SAMP8 mice [27]. In this study, a trend toward a decrease in the amount of APP in HPBR-fed mice was observed (Fig. 5c). This might be one of the reasons why HPBR reduced the amount of Aβ. The HPBR-fed mice had better motor functions than the control and PR-fed mice. The HPBR-fed mice had a significantly greater grip strength than the control mice (Fig. 4b). Also, in rotarod test, the HPBR-treated mice had a trend toward an increase in fall latency (Fig. 4a). The HPBR-treated mice also had a higher total number of arm entries in the Y-maze than those in the other groups (Fig. 3b). Motor dysfunction may be correlated with cognitive dysfunction in AD or other dementias. Middle-aged adults with a slow walking speed and weak hand grip have a more than 2.5-fold increased risk of developing AD [33]. Reduced muscle strength or motor activity leads to decreased blood flow in the entire body, including the brain. Decreased cerebral blood flow results in lack of oxygen and nutrition, which is associated the deterioration of neurological function. Improvement in motor function by associated with HPBR may thus contribute to the prevention of AD and other dementias. Previous studies have evaluated the effects of BR on diabetes or obesity [39, 40]. BR is rich in dietary fiber which blocks the absorption of sugar and fat from the gastrointestinal tract and contributes to its beneficial effects in diabetes or obesity. However, there are few reports on the effects of BR on dementia in animal models or in humans. The nutrients in BR may be difficult to absorb, and HPBR may have ameliorated cognitive dysfunction in SAMP8 mice because HP treatment made BR easier to digest.

The adoption of daily activities that help prevent AD and other dementias is important, and dietary strategies are realistic and practical. We believe that HPBR offers suitable benefits and effects against AD and other dementias in human and that this topic requires further investigation.

## Conclusions

HPBR improved cognitive function and decreased Aβ levels in the brain of the SAMP8 mice, suggesting that it is useful for preventing AD.

## Abbreviations

AD: Alzheimer’s disease
ANOVA: analysis of variance
APP: amyloid precursor protein
Aβ: amyloid β
BR: brown rice
FA: ferulic acid
GABA: gamma-aminobutyric acid
HP: high-pressure
HPBR: highly water pressurized brown rice
PR: polished rice
SAMP8: senescence-accelerated mouse prone 8
